# Cells and Cell Life

**Published:** 1873-11

**Authors:** Ch. Rauschenberg


ATLANTA

MEDICAL AND SURGICAL JOURNAL.

VOL. XI.] NOVEMBER—1873. [No. 9.

### Original Communications.

### CELLS AND CELL LIFE.*

### READ BEFORE THE HISTOLOGICAL AND PATHOLOGICAL SOCIETY OF ATLANTA.

BY CH. RAUSCHENBERG, M.D.

EVERY important revolution and every material progress in the pathological knowledge of the different epochs in the history of medicine has been preceded and initiated by anatomical discoveries, revealing previously unknown facts in relation to the organization and structure of the human body. The first system of medicine, that of Galen, in the first centuries of the Christian Era was already based upon the imperfect anatomical ideas of the Alexandrian School, and when medical science, after the long continued tethargy of all mental pursuits during the middle ages, began to revive again, it received its principal stimulus by the labors of Vesal, (1845–65), the founder of anatomy, and the anatomical discoveries of Harvey, (1576–1679), Aselli, Malphigi, and others. When Bichat at the commencement of this century made the first effort to establish a system of general anatomy by

* This article was not intended for publication by its writer, but merely for the promotion of the object of the Society, before which it was read, to-wit: "Mutual Instruction." It is based principally upon the information on this subject contained in the following works, which have been freely used in its composition: Handbuch der Anatomie des Menschen, von Dr. C. E. Bock, Leipzig, 1842; Der Einfluss der Cellular-Pathologie auf die ærztliche Prasis, von Dr. C. A. W. Richter, Berlin, 1863; von Prof. Dr. R. Virchow, 1871; and a Manual of Hstiology Die Cellular-Pathologie, von Prof. S. Stricker, translated by Henry Power and others, New York, 1872.

dividing and classifying the tissues of the human system according to their physical, chemical and vital qualities with a view of arriving at a knowledge of certain elementary tissues, endowed with specific vital qualities, he created a revolution in medical thought and investigation, which is still in operation, and which is changing the very principles of medicine and threatening a radical reform of entire system of the medical conceptions.

Richerand, Cloquet, Meckel, Rudolphi, Wagner and others became his followers and made modifications and improvements in his classification of the tissues, as their anatomical knowledge of the different structures increased by microscopic investigations.

With the great improvements in the construction of the microscope and the increase of the technical skill in its use, the progress in minute anatomy, and its influence on physiology and pathology, assumed dimensions unexcelled in the history of medicine by any previous discoveries or methods of investigation.

The microscopical search after the elementary constituents of the different tissues, introduced by Bichat, led gradually to more definite results. Treviranus, in 1816, established three different kinds of anatomical elements: homogenous but amorphous substance, globules and cylinders, or fibres, by the mutual coaptation of which all tissues are formed. Raspait, in his investigations of the microscopic structure of starch and fat, first represents the organic atoms or molecules of these substances as vesicles, endowed with vitality, and with the faculty to produce within themselves, *ad infinitum*, new vesicles of the same structure and with the same vital faculties. He compares them as the atoms of organic creation with crystals and calls organization, or organic development, a process of crystalization in vesicles, occasioned by introsusception—and exclaims: "Give me a vesicle capable of filling itself by suction, and I will make you an organism." Dutrochet arrived at similar conclusions. He takes the solid parts of the body to be aggregates of cells of a certain consistency, and the fluid parts aggregates of cells kept apart by fluids. The only solid parts of organic substance are, with him, the cell-membranes; and active vitality in the cell continues, with him, only as long as its contents are fluid.

Robert Brown discovered, in 1831, in the vegetable cells, what he called the areola—the nucleus of later observers—and what the German botanist, Schleiden, soon afterward proved to be the most important organ in the development of the cell. He was the first one who established definite views in relation to the growth and reproduction of the vegetable cell.

Isolated observers had, previous to Schleiden's discoveries, discovered similar nucleated vesicles in the animal organism, without, however, arriving at a comprehension of their importance. Thus the red corpuscles of the blood, the corpuscles of the lymph, of mucus, of pus, of the epithelium, of different glands, and of some pathological products, were known before the appearance of Schleiden's work; and Purkinge, Valentine, and Turpin had called attention to their similarity with the vegetable cell. Valentine had observed the pre-existence of the nucleus and the gradual growth of cells on the cells of the pigment, Schultze on the blood corpuscles, Wagner on the ovum, Henle on the epidermis; but it was Schwan who laid the foundation to a systematic classification of the animal tissues, by establishing the fundamental principle *that nucleated cells are the basis of all vegetable and animal organization.* He first discovered that the so-called cytoblastem, amorphous substance contained within or around the cell, constitutes the material within which cells develop and multiply according to definite laws, and form the elementary tissues of the organism.

These discoveries of Schleiden and Schwan, followed by the investigations of Valentine, Henle and others, soon led to a full comprehension:

1. Of the specific vitality of the organic cell as an independent individuality, which has its time of birth, its period of life, and time of death; which, during its mature life, discharges specific functions, but in doing so wears out and decays.

2. That all tissues primarily owe their origin, development, and specific character, to the specific vital powers of their cells; and

3. That alike physiological phenomena or the same functions are attached to organs of the same structure, or, in other words, to elementary parts of the same morphological and chemical composition.

Virchow, however, went still further. He looks upon the cell as the starting point of all phenomena of life, and, therefore, upon the knowledge of its activity as the basis of all physiological and pathological conceptions in health and in disease. *The cell, with him, is the true organic unit, the primary form ele-*

*ment of all organization, in health and in disease, from which all vital action originates—the primary seat of life.*

The present views in relation to the nature and vital qualities of cells—after thus giving a condensed history of histology and its influence on medical science—occupy our attention next.

In consequence of the fact that the earlier investigations of cell structure were principally made on plants, and that similar formations were observed in the tissues of the animal body, the conception became universal amongst histologists that every cell, in its perfect development, represented a vesicle surrounded by a cell-membrane forming a cavity, filled with substance of some kind, called formerly cytoblastem, now protoplasma, and nucleus. Later investigations have demonstrated that the supposed identity of structure between the vegetable and the animal cell, at least as Schwan accepted it, is to some extent erroneous. The exterior cellulose membrane of the vegetable cell as the product of acquisition from without, due to subsequent development, has nothing in common with any part of the animal cell. It consists of carbonaceous material entirely, while all the other part of the elementary cells of animals and plants contain only nitrogenous material. When we divest the vegetable cell in our mind of that exterior membrane, we arrive again at a correct conception of the uniform and apparently monotonous formation, whose constant and invariable occurrence in all living organisms betokens its truly elementary character as the source of all vital progress and development. The cell membrane of the animal cell corresponds, therefore, with the inner membrane of the vegetable cell, the primordial sac, as Hugo von Mohl calls it.

These rather ideal conceptions of the structure of the animal cell as consisting of a vesicle of variable shape, defined by a cell membrane, and possessing a nucleus and a fluid protoplasma within its cavity, have in course of time undergone some changes.

Leydig (1845) abandoned this scheme of cell construction, and maintained that the contents of the cell are of higher dignity than the membrane, and that the conception of a cell requires only the presence of a little mass of protoplasma enclosing a nucleus. This cell membrane, in his view, is only the hardened external layer of the cell substance. Max Schultze takes the embryonal cells, which proceed from the division of the cells of the ovum, as the pattern of his conceptions on cell structure.

He sees in them the archtype of a cell, and they consist only of nucleated protoplasma granules. He also demonstrated that the contractile substance which Dujardin discovered in 1835 in the very lowest animal organizations, is identical with the protoplasma of cells. As Dujardin found this substance, although destitute of nerves, endowed with the power of contraction and of motion, and therefore possessed of irritability, these granules of sarcode, as he called them, were considered living and independent organisms. This consideration imparted a peculiar physiological significance to Schultze's declaration that it and the protoplasma of animal cells were one and the same specific organic elementary substance.

Under the influence of these and other observations too numerous to detail in this paper, the above mentioned conclusions in relation to the structure of the animal cell underwent certain modifications which are condensed into the following statements:

1. The so-called cell-membrane of the animal as well as vegetable cell, is not any longer considered an indispensable requisite to constitute a cell. *A nucleus surrounded by protoplasma forming a soft but still solid and defined body, is sufficient to justify the application of that term.* Only at a certain state of progressive development, the formation of a cell membrane takes place.

2. The fluid contents of the cell—the protoplasma—is the most productive and the most essential part of the cell. The specific vital qualities of single cells and cell groups, and the character of the intercellular substance, which they produce; hence the physiological character of the tissues which they form depends upon the characteristic peculiarities of the protoplasma, or the cell contents of which they originally consist.

3. The cell nucleus, though its existence may not be an indispensible necessity in the cells of some of the lowest forms of vegetable and animal life, is that part of the cell which maintains its uniformity of shape in all kinds of cells more constantly than any other part of the cell as long as the cell exists in its full vitality, and the specific integrity of each class of cells seems to be prominently attached to the existence of the nucleus. Wherever the nucleus is wanting in a cell, that cell has already lost its specific individual type, and becomes a structure subject to changes and variations. Thus the red blood corpuscles, after losing the nuclei which they possess during a portion of intra-uterine life, become elements which are not intended to enjoy a durable existence, but to undergo frequent changes and early decay. Thus also we find no nuclei in the upper layer of the epidermis. Upon the same principal, it is true that no material physiological and pathological changes of cells take place but what are principally and prominently indicated by alterations in the shape of their nuclei.

Thus it happens that the nucleus plays an important role in the life of the cell; a role which is not so much connected with the functional activity of the cell as with the maintainance and multiplication of its elements as living parts. In all tissues which grow physiologically or pathologically, nucleated elements are always the starting points of the first changes. The nucleoli contained in the nucleus are not an indispensible element of the cell. In many young elementary cells they cannot be discovered, while they occur regularly in well developed older cell-formations, seemingly indicating a higher perfection of the cell.

Thus the definition of a cell has been considerably enlarged, and in modern histology a minute granule of protoplasma without a nucleus would be denominated a cell, as soon as it exhibited the entire group of phenomena which are characteristic of an independent organism.

The shape of different cells varies as much as the nature of their contents. The cells of the blood, the lymph, the mucus, the pus, the fat, the pigment, the liver, etc., are of a more or less regularly round or oval form; the connective tissue contains elongated, cylindric, or spiral cells; the lymphatic glands stellated cells, and the ganglia cells of an irregular bifurcated shape, all with nuclei of the same uniform appearance. Some cells contain pigment, some fat, some contractile substance, as the muscle cells, etc.

The physiological qualities of the protoplasma, when it makes its appearance as a cell, are active or spontaneous movement, nutrition, growth, and reproduction—all the essential qualities of individual life.

Movements of cells may be easily seen under the microscope. The growth of the cell is a more gradual process and cannot be observed; nor has any one been able to place cells under the microscope under such favorable conditions as to witness their increase in size. We must, therefore, be led by analogy to the conclusion that this does take place. Various observations have been directly made on the nutrition of unicellular animals,

(amœba). It can be seen how they take up foreign bodies and nutrient material into their bodies, and some of the changes of the material introduced may be noticed.

The act of reproduction, depending upon the growth of the mother and detachment of the daughter cell is, at least as far as the latter part of the process is concerned, often enough seen under the microscope.

Schleiden and Schwan established the theory of a spontaneous formation of cells from homogenous particles floating in the cytoplastem, the plastic material of the organism. These particles were to put on development in a mysterious manner, form nucleoli, and then by their aggregation nuclei, from which again the cell-membranes and their contents were to develop themselves. This theory of spontaneous cell development has not been sustained by the observations of later histologists. *All organic material receives the faculty of organization by being absorbed and influenced by the specific action of the cell; all plastic material is contained principally within or has passed through the cell and organization without a mother cell, development de novo, spontaneous generation so far is considered an untenable hypothesis.* Wherever a cell is formed, another cell has preceded it, as much so as an animal can only originate from an animal, a plant only from a plant. This fact has given rise to Virchow's aphorism: "Omnis cellula e cellula," in correspondence with the older, "omne vivum ex ovo."

The reproduction of cells from mother cells takes place in three different ways: (1) By fissure, (2) by budding, (3) by formation of young cells within the mother cell. Each process is easily comprehended, and can occasionally be observed under the microscope. In the first case, the mother cell shows a circular constriction, which, by becoming narrower and narrower, divides the mother cell, its wall, contents, and nucleus, by hourglass contraction into two young cells. In the second case, the mother cell sends out a nucleated process, which gradually becomes larger, and finally separates itself; and in the last case, the so-called endogenous generation, an indefinite number of young cells with their own membranes and nuclei develop themselves within the mother cell, and escape from their confinement either by fissure or deliquescence of the walls of the mother cell.

Therefore, cells must be recognized as independent organized individualities, and as the primary elements of organic life, to which all parts and tissues of organized beings owe their origin, growth, and specific character. Upon the typical peculiarity and the specific vital action of the formative cells depends the specific character of the different tissues.

The vegetable cell can only germinate under the influence of certain external conditions. It needs moisture, warmth, and suitable nutrient material. The animal cell needs similar external influence for its development, and what the supporting substances of the soil are to the former, that is the blood and the lymph, which permeate all parts and particles of the organism to the latter. They constitute the nutritive fluid. The specific cell selects for its nourishment, growth and reproduction, certain substances from them, appropriates them by endosmosis and converts them into new cells and secretes plastic material, intercellular substance, around itself in a certain order and proportion, and thus by its specific action originates and developes the different specific tissues of the organism.

During the formation of the different materials and tissues of the animal organism the elementary cell undergoes manifold changes. Certain cells remain isolated and single, only changing their form, contents and chemical composition ; others lose their single individuality by melting together into secondary cells of various form. Some become solid, some retain their cavity filled with clear homogenous or with more or less granular fluid, some still exhibit a nucleus, some a longitudinal arrangement of their nuclei into cylinders or fibres, some lose them entirely, etc.

The different metamorphoses of the elementary cell may be arranged under the following scheme :

I. *The elementary cells retain their individuality.* Cells, which never unite with each other but only undergo changes in form, contents and chemical composition, sometime retaining, sometime losing their nucleus. They expand and grow either uniformly in all directions and retain their globular form or where they touch each other become polygonal or grow principally in one direction, vertically or longitudinally, and represent plates, scales, conical, prismatic or cylindric corpuscles. Their contents change in consistency from the fluid to the granular and solid state, and from the latter to the former, and while, when young, their cell membrane is soluble in acetic acid, at a later period of their existence this is not the case.

These cells make their appearance in the perfected animal organism:

(*a*) As isolated cells suspended in fluids.—They do not form tissues, remain isolated floating cells and retain their original form and nucleus. Such cells are lymph corpuscles, the blood corpuscles, mucus corpuscles, and pus corpuscles.

(*b*) As isolated cells promiscuously scattered about in other elementary tissues:—Fat cells, pigment cells, parenchyma cells, ganglionic cells.

(*c*) As isolated cells retaining their membranes and nuclei in continuous apposition to each other, forming tissues without fusing into each other:—Some retain their globular forms, some become flat and polyedric, others cylindric or conical. The epithelial cells of the mucous membranes, the skin, the hair, the nails, the prismatic cells of the enamel of the teeth. The isolated fibres of the crystaline lens belong to this class.

II. *The elementary cells lose their individuality.* Neighboring cells fuse with each other; the separating cell walls, if such exist, are absorbed, disappear, and their cavities unite into one. This second class of cells is again sub-divided into two great groups:

*A.* The fusing elements are cells fully developed consisting of more or less solid cell membranes and contents.

This fusion takes place—

1. Between the cell membranes while their cavities remain separated: cartilage cells, bone corpuscles, corpuscles in the substance of the teeth.

2. Between the cell cavities by resorption of the dividing wall partitions. This process appears in three different forms:

(*a*) The cells are longitudinally attached to each other and convert themselves into continuous tubes, muscular fibres, nerve tubes.

(*b*) The cells are arranged around a common center and by resorption of their wall segments form grape-shaped cells: acini of the glands.

(*c*) The cells send out hollow processes in different directions which meet other processes from neighboring cells, and fuse with them into a net of more or less longitudinal tubes. The capillary vessels and the stellated pigment cells are thought to originate in this manner.

*B.* The fusing elements are cells without membranes or protoplasma granules, which are converted into minute fibrillæ and bundles of fibrillæ, or into layers of transparent hyaline membranes and the nuclei into fibrillæ which is found belonging to each bundle of protoplasmatic or cell fibrillæ. The former are soluble; the latter not soluble in acetic acid—proving their origin respectively from the one or the other. These formations are represented in the tissues of the cornea, the lens and its capsule. Descemets membrane, in the exterior membrane of the nerve tubes and the animal muscle fascicle, in the muscular membrane of the blood vessels and intestines, in the bone of the teeth and their enamel, in the cordical substance of the hair, etc.

This rather abstract scheme represents Henle's ideas of the processes by which the principal form-elements of the animal organization arrive at their spêcific configurations. While later histological discoveries have changed many of its details, its general features still afford an elementary basis for histological conceptions and form an introduction into that science; on that account I thought it proper to give you its outlines in this paper.

[TO BE CONTINUED.]

